# The Inflammatory Marker YKL-40 Is Elevated in Cerebrospinal Fluid from Patients with Alzheimer’s but Not Parkinson’s Disease or Dementia with Lewy Bodies

**DOI:** 10.1371/journal.pone.0135458

**Published:** 2015-08-13

**Authors:** Malin Wennström, Yulia Surova, Sara Hall, Christer Nilsson, Lennart Minthon, Oskar Hansson, Henrietta M. Nielsen

**Affiliations:** 1 Clinical Memory Research Unit, Department of Clinical Sciences Malmö, Lund University, Malmö, Sweden; 2 Department of Neurology, Skåne University Hospital, Lund, Sweden; 3 Memory Clinic, Skåne University Hospital, Malmö, Sweden; 4 Department of Neuroscience, Mayo Clinic College of Medicine, Jacksonville, Florida, United States of America; University of Barcelona, SPAIN

## Abstract

A major difference in the revised diagnostic criteria for Alzheimer’s disease (AD) is the incorporation of biomarkers to support a clinical diagnosis and allow the identification of preclinical AD due to AD neuropathological processes. However, AD-specific fluid biomarkers which specifically distinguish clinical AD dementia from other dementia disorders are still missing. Here we aimed to evaluate the disease-specificity of increased YKL-40 levels in cerebrospinal fluid (CSF) from AD patients with mild to moderate dementia (n = 49) versus Parkinson’s disease (PD) (n = 61) and dementia with Lewy bodies (DLB) patients (n = 36), and non-demented controls (n = 44). Second we aimed to investigate whether altered YKL-40 levels are associated with CSF levels of other inflammation-associated molecules. When correcting for age, AD patients exhibited 21.3%, 27.7% and 38.8% higher YKL-40 levels compared to non-demented controls (p = 0.0283), DLB (p = 0.0027) and PD patients (p<0.0001). The AD-associated increase in YKL-40 was not associated with CSF P-tau, T-tau or Aβ42. No relationship between increased YKL-40 and levels of the astrocytic marker glial-fibrillary acidic protein (GFAP), interleukin-8 (IL-8), monocyte chemoattractant protein-1 (MCP-1) and interferon gamma-induced protein 10 (IP-10) could be identified. Our results confirm previous reports of an age-associated increased in CSF YKL-40 levels and further demonstrate increased CSF YKL-40 in AD patients versus non-demented controls and patients with DLB or PD. The increase in YKL-40 levels in the AD patients was unrelated to the established CSF AD biomarkers and the inflammatory markers GFAP, MCP-1, IP-10 and IL-8, proposing YKL-40 as a marker of yet to be identified AD-related pathological processes.

## Introduction

Alzheimer’s disease is the most common cause of neurodegenerative dementia with a prognosis of dramatically increased disease prevalence. In the USA alone the number of deaths as a result of AD was 83,494 individuals in 2010 and the number of afflicted people has been estimated to increase from 4.7 million in 2010 to 13.8 million in 2050 [1]. Based on the increasing understanding of AD development and progression, new diagnostic and research criteria were proposed a few years ago [2–4]. The revised guidelines outline three different AD disease stages; preclinical AD, mild cognitive impairment (MCI) due to AD and AD with dementia. A major difference from the previous diagnostic criteria is also the incorporation of biomarkers which in addition to supporting a clinical diagnosis also allow the identification of preclinical AD and mild cognitive impairment (MCI) due to AD neuropathological processes including Aβ accumulation and neurodegeneration. A biomarker is defined as ‘a characteristic that is objectively measured and evaluated as an indicator of normal biological processes, pathogenic processes, or pharmacologic responses to a therapeutic intervention’ as proposed by the Biomarkers Definitions working group [5]. Currently widely used fluid biomarkers to identify Aβ deposition and neuronal injury include cerebrospinal fluid (CSF) biomarkers; low Aβ levels reflect increased Aβ deposition and increased levels of phosphorylated tau reflect neurofibrillary tangle formation. However not only AD patients exhibit altered levels of tau and Aβ, these features are also frequently described in other disorders like Creutzfelt-Jakob disease, dementia with Lewy bodies (DLB), frontotemporal dementia (FTD) and vascular dementia (VAD) [6]. Thus, specific AD biomarkers which accurately aid the detection of pathological AD processes and subsequently distinguish clinical AD dementia from other dementias, are still missing. Interestingly, several recent studies have separately identified increased levels of the astrocytic marker YKL-40 also called chitinase-3-like 1 (CHI3L1) in CSF from AD patients [7–10]. The glycoprotein YKL-40 is up-regulated in various inflammatory conditions and expressed by different cell types including activated neutrophils, macrophages, chondrocytes, synoviocytes and vascular smooth muscle cells [11]. In the brain, YKL-40 is mainly expressed by astrocytes and its protein levels were reported to be elevated in both acute neurological disorders like traumatic brain injury and stroke as well as in chronic disorders like amyotrophic lateral sclerosis (ALS) and multiple sclerosis (MS) [12, 13]. Increased CSF YKL-40 concentrations were previously also reported in patients with preclinical, very mild or mild AD [7] and in individuals with mild cognitive impairment (MCI) and an AD-indicative AD biomarker profile when compared to stable MCI patients with a normal AD biomarker profile, and healthy controls [8]. Furthermore, evidence supporting an association between AD pathology and YKL-40 was found using immunohistochemical stainings showing YKL-40 reactive astrocytes in close proximity to amyloid plaques [7]. In the current study we aimed to quantify and compare CSF levels of YKL-40 in AD patients compared to patients with other neurodegenerative disorders i.e. dementia with Lewy bodies (DLB) and Parkinson’s disease (PD). In order to investigate whether YKL-40 is associated with secretion of other inflammatory markers, we also quantified CSF levels of the glial fibrillary acidic protein (GFAP), a marker for astrocyte activation, and a panel of pro-inflammatory chemokines in AD patients and non-demented controls.

## Materials and Methods

### Study subjects

A total of n = 190 individuals were included in the study whereof n = 44 were non-demented non-demented controls, n = 49 were clinically diagnosed with AD, n = 36 with DLB and n = 61 with PD. Patients and control subjects were seen in the Neurology and Memory Clinics at Skåne University Hospital, Sweden, and are a sample from an earlier described cohort [14, 15]. Patients with an AD diagnosis met the DSM-IIIR criteria for dementia (American Psychiatric Association 1987) and the probable AD criteria defined by NINCDS-ADRDA [16], patients diagnosed with DLB met the consensus criteria [17] and patients with PD met the NINDS Diagnostic Criteria for Parkinson’s Disease [18]. The non-demented controls were individuals clinically evaluated for subtle cognitive symptoms. After a thorough diagnostic dementia work-up they were judged to be cognitively healthy. Study subjects were sampled non-fasting at no particular time of the day and in line with the recommendations outlined in the Alzheimer’s Association Flow Chart for LP and CSF sample processing[19]. All individuals underwent clinical examination including a CSF AD biomarker (Aβ1–42, T-tau and P-tau) and CSF α-synuclein assessment using commercial ELISA assays (Innogenetics and Invitrogen) as previously described [14, 15]. Hence, CSF samples were collected in polypropylene tubes, centrifuged at 4°C at 2000x g for 10 min within 30 min after collection. Samples were then aliquoted and stored at -80°C pending biochemical analysis. All individuals gave informed consent either by use of a passive consent procedure where consent for retrospective use of banked clinical samples and data was assumed if individuals did not actively retract permission, as instructed in repeated local press advertisements, or by active written informed consent. Informed consent to use samples drawn as part of the clinical routine examination (no samples were drawn for the sole purpose of research) was documented in two separate registries including the patient medical chart and in the local clinical research database. The study protocol and the manners to consent were approved by the local ethics committee at Lund University, Sweden, and conducted in agreement with the Helsinki Declaration.

### Analysis of CSF YKL-40 and GFAP

Cerebrospinal fluid levels of YKL-40 and GFAP were quantified using commercially available sandwich enzyme linked immuno-sorbent assays (ELISAs). The detection limit of the YKL-40 ELISA (R&D Systems) was 2.32 pg/mL with an intra- and inter-assay coefficient of variation (CV%) of 5% and 8% respectively. Recovery of spiked YKL-40 concentrations into diluted CSF samples was 94% (55%-118%). The detection limit of the GFAP ELISA (Biovendor) was 0.023 ng/mL with intra- and inter-assay CV% of 12% and 7%, respectively, and 100% (82%-113%) recovery. The optical density at 450/540 nm was determined using a microplate reader (Labsystems iEMS) using background correction. Readings of standards and samples for each assay were averaged and the concentrations of YKL-40 and GFAP determined by use of a 4 parametric curve fit. To minimize and control for potential bias from inter-assay variation, samples from all diagnostic groups alongside two internal controls of pooled CSF samples were included in each assay

### Analysis of additional inflammatory markers in CSF from AD patients and non-demented controls

Concentrations of inflammatory markers in CSF from non-demented controls and AD patients were determined using a Human Chemokine 9-Plex ultra-sensitive electrochemiluminescence immunoassay (MesoScale Discovery, UK). The MSD 9-plex immunoassay was designed for the analysis of Eotaxin, Eotaxin3, IL-8, Interferon gamma-induced protein 10 (IP-10), MCP-1, Monocyte chemotactic protein-4 (MCP-4), Macrophage derived chemokine (MDC), Macrophage inflammatory protein 1β (MIP1β) and Thymus activation-regulated chemokine (TARC), however only MCP-1, IL-8 and IP-10 levels were within detection range of the assay. The detection limits for IL-8, MCP-1 and IP-10 were 0.43, 16.20 and 8.48 pg/mL. Intra- and inter-assay variations (CV%) for IL-8, MCP-1 and IP-10 were 7%, 27% and 9%, and 8%, 7%, and 23% respectively. Each standard and sample was analyzed in duplicates and resulting electrochemiluminescence was quantified using an MSD SECTOR Imager 6000. Duplicate readings of standards and samples were averaged and the concentrations of individual markers were determined by interpolation from individual standard curves using a 4-parametric curve fit. As described above, samples from all diagnostic groups were included in each assay in order to avoid inter-assay variation bias.

### Statistical analyses

Statistical analysis was performed using the JMP Pro 10.0.0 software. Normal distribution was assessed by use of the Shapiro-Wilk W test. Log-transformation was used to enhance normal distribution of CSF YKL-40 and GFAP data. Log-transformed data was compared between investigated groups using ANCOVA with age entered as a covariate and subsequent posthoc-testing was performed using Bonferroni correction. Differences in CSF T-tau, p-tau and Aβ1–42 were analyzed using the non-parametric Kruskal-Wallis test and when significant followed by pair-wise comparisons using Wilcoxon with Bonferroni correction for multiple comparisons. The non-parametric Mann-Whitney U test and ANCOVA with age entered as a covariate, were used to assess differences in CSF inflammatory marker levels between non-demented controls and AD patients. Correlations were investigated using multivariate regression and the Spearman’s Rank correlation test. The performance of elevated CSF YKL-40 levels as a discriminating marker for AD against the other included diagnostic groups was evaluated using receiver operating characteristics (ROC) curves. Results are presented as means or medians with either standard deviation or range. A p<0.05 was considered significant.

## Results

### Clinical characteristics

Complete demographic data and available AD biomarker profiles describing the included study subjects have been reported earlier [14, 15]. Updated and relevant data for the herein included subjects are summarized in Table 1 (specific distribution of the individual markers in the AD biomarker profiles are shown in S1A–S1D Fig). Whereas AD and DLB patients exhibited MMSE scores indicative of mild to moderate dementia, PD patients exhibited MMSE scores only slightly lower than non-demented controls (p<0.05). Non-demented controls were significantly younger than AD, DLB and PD patients. The AD group had a significantly different gender distribution with more females compared to non-demented controls. Also, similar to AD patients, individuals diagnosed with DLB had decreased levels of CSF Aβ1–42 compared to non-demented controls (p<0.001) however patients with DLB and PD did not differ from non-demented controls in respect to CSF T-tau or P-tau levels.

**Table 1:** Study subject characteristics.

Diagnosis	Age (yrs)	Gender (% M/F)	MMSE score	CSF T-tau (ng/L)	CSF P-tau (ng/L)	CSF Aβ1–42 (ng/L)	APOEε4 carriers (%)
**Non-demented** *Total N = 44*	63.7±10.3	48/52	29±1	483	49	585	33
				(149–1952)	(23–115)	(259–994)	
				(N = 35)	(N = 39)	(M = 39)	
**AD** *Total N = 49*	77.1±6.0***	24/76	20±4	898***	105***	361***	76
				(174–2472)	(42–175)	(131–633)	
				(N = 38)	(N = 43)	(N = 47)	
**DLB** *Total N = 36*	74.6±5.7***	43/57	21±5	586	58	424***	52
				(264–1040)	(17–115)	(180–754)	
				(N = 21)	(N = 34)	(N = 34)	
**PD** *Total N = 61*	68.4±9.2*	59/41	27±3	371	48	582	ND
				(120–862)	(42–74)	(261–850)	
				(N = 48)	(N = 52)	(N = 52)	

***) indicates a difference compared to non-demented controls, p<0.001

*) indicates a difference compared to non-demented controls <0.05, (ND) not determined.

### YKL-40 levels are increased in AD patients

**Fig 1:**
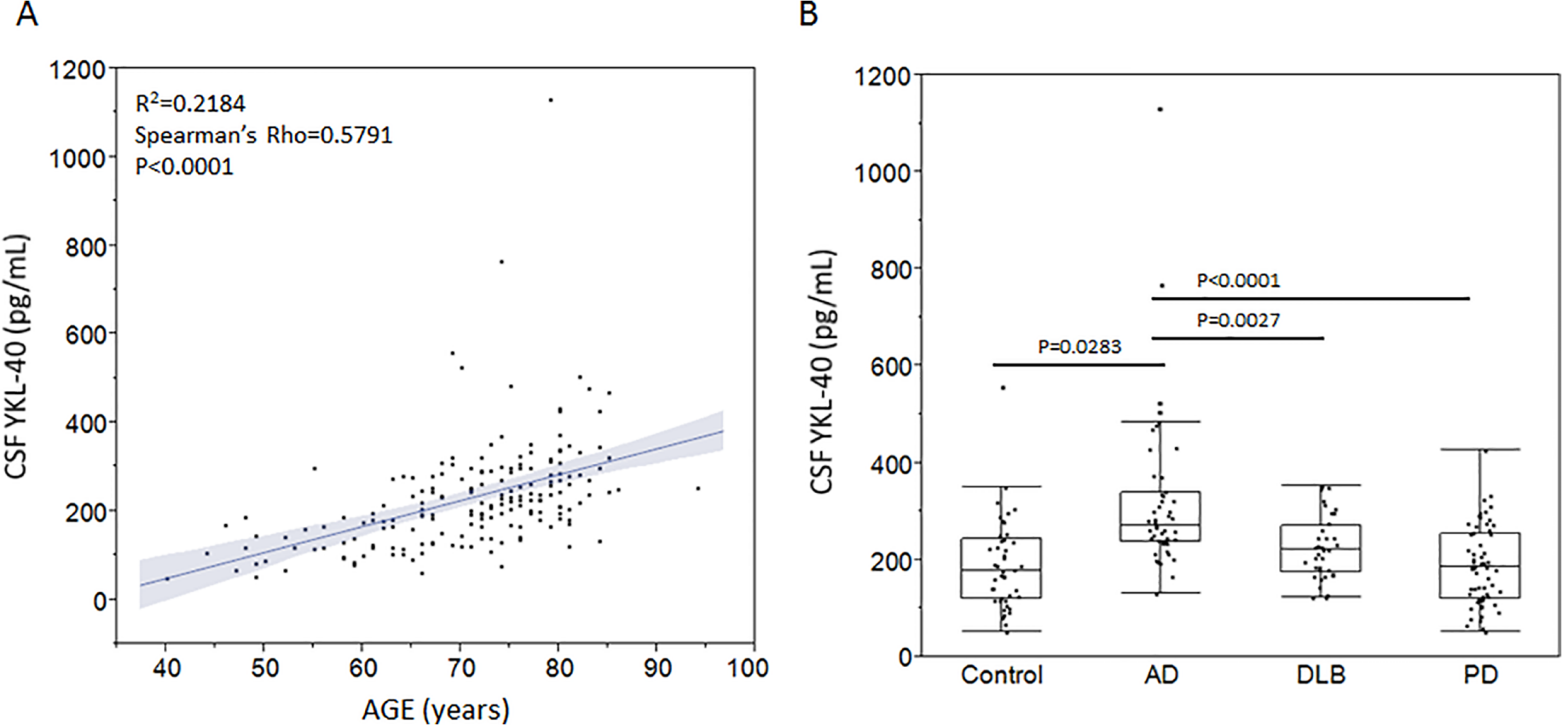
Cerebrospinal fluid YKL-40 levels are linked to age and AD. Cerebrospinal fluid concentrations of YKL-40 (A) were positively and significantly associated with age as determined using the Spearman’s Rho non-parametric correlation test (A). Increased CSF YKL-40 levels were found in AD patients compared to all other investigated groups as assessed wit log-transformed data and the ANCOVA test with age entered as a covariate (displayed data are unadjusted). Subsequent posthoc-testing was performed using Bonferroni correction.

As previously reported [7], CSF levels of YKL-40 were strongly associated with age (Spearman’s rho = 0.5791, p<0.0001) with increasing age leading to higher levels of this marker (Fig 1A). When subsequently adjusting the group comparisons using age as a covariant, increased levels of YKL-40 were found in AD patients compared to all other patient groups and non-demented controls (ANCOVA F ratio 6.8535, p = 0.0002). Patients with AD exhibited mean CSF YKL-40 concentrations that were 21.3%, 27.7% and 38.8% higher than those of non-demented controls (p = 0.0283), DLB (p = 0.0027) and PD patients (p<0.0001) respectively (Fig 1B). The area under the curve (AUC) values generated by ROC curve analyses to assess the performance of CSF YKL-40 as an AD marker to discriminate this group from non-demented controls, DLB and PD patients were 0.816, 0.736 and 0.818 respectively. We found no effect of gender or APOEε4 status on YKL-40 levels (data not shown).

### Levels of IL-8, IP-10 and MCP-1 and GFAP are associated with age and not increased in AD patients

**Fig 2:**
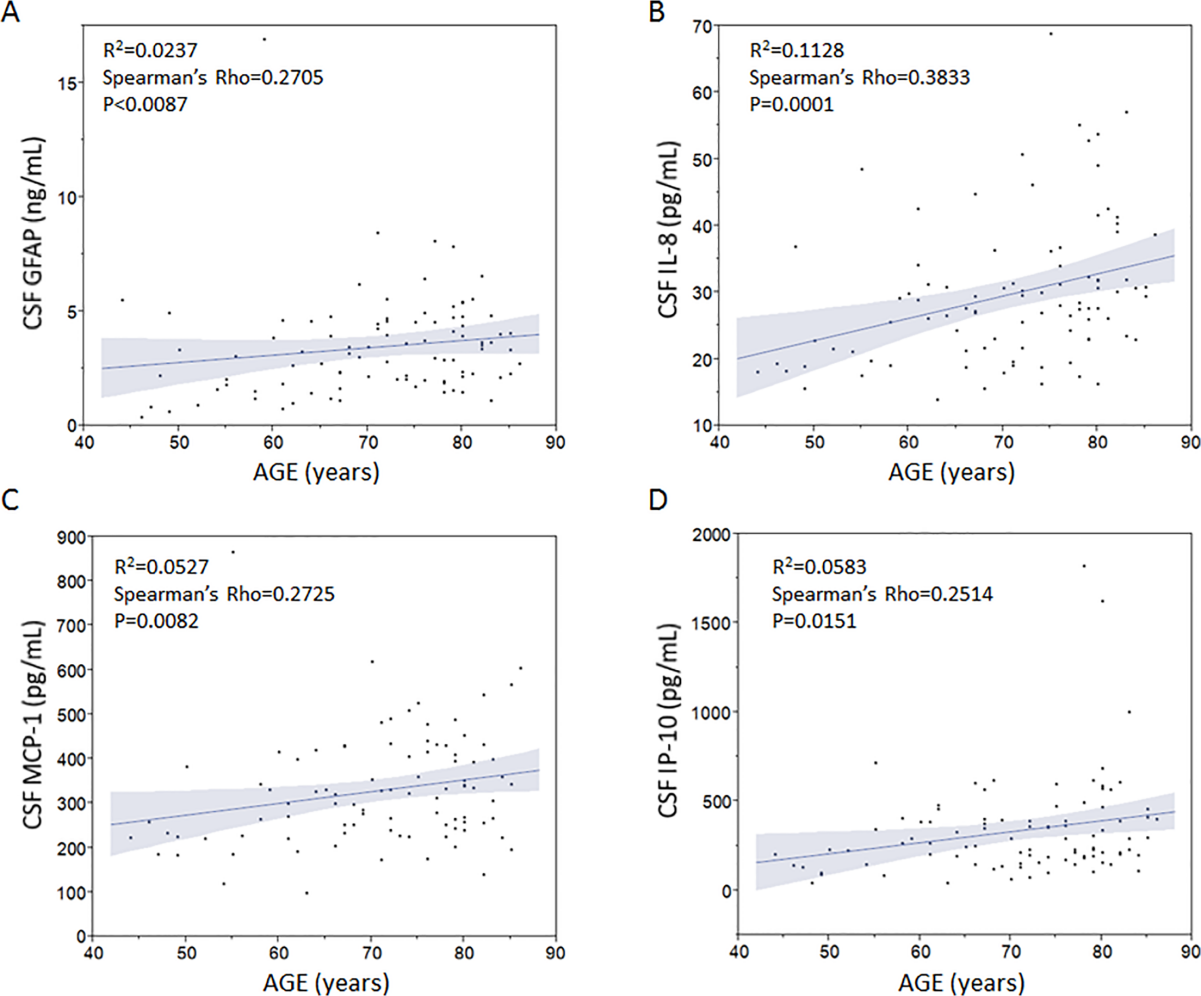
Levels of inflammatory markers in cerebrospinal fluid are linked to age. Cerebrospinal fluid levels of proinflammatory markers including GFAP (A), IL-8 (B), MCP-1) (C), IP-10 (D) were weakly but significantly correlated to age as determined by the non-parametric Spearman’s Rho correlation test.

In order to further interrogate whether the observed AD-related increase in YKL-40 levels is associated with other inflammatory markers, we employed an ELISA to quantify CSF GFAP levels and in addition we used an MSD multiplexed cytokine/chemokine panel to quantify pro-inflammatory markers in CSF from AD patients versus non-demented controls. Similar to YKL-40, CSF levels of GFAP were weakly but positively linked to age (Fig 2A). Further, out of the nine investigated inflammatory markers included in the multiplexed panel, only n = 3 markers i.e. IP-10, IL-8 and MCP-1 exhibited detectable and quantifiable levels in CSF. All of these three markers were also positively associated with age (Fig 2B–2D).

**Fig 3:**
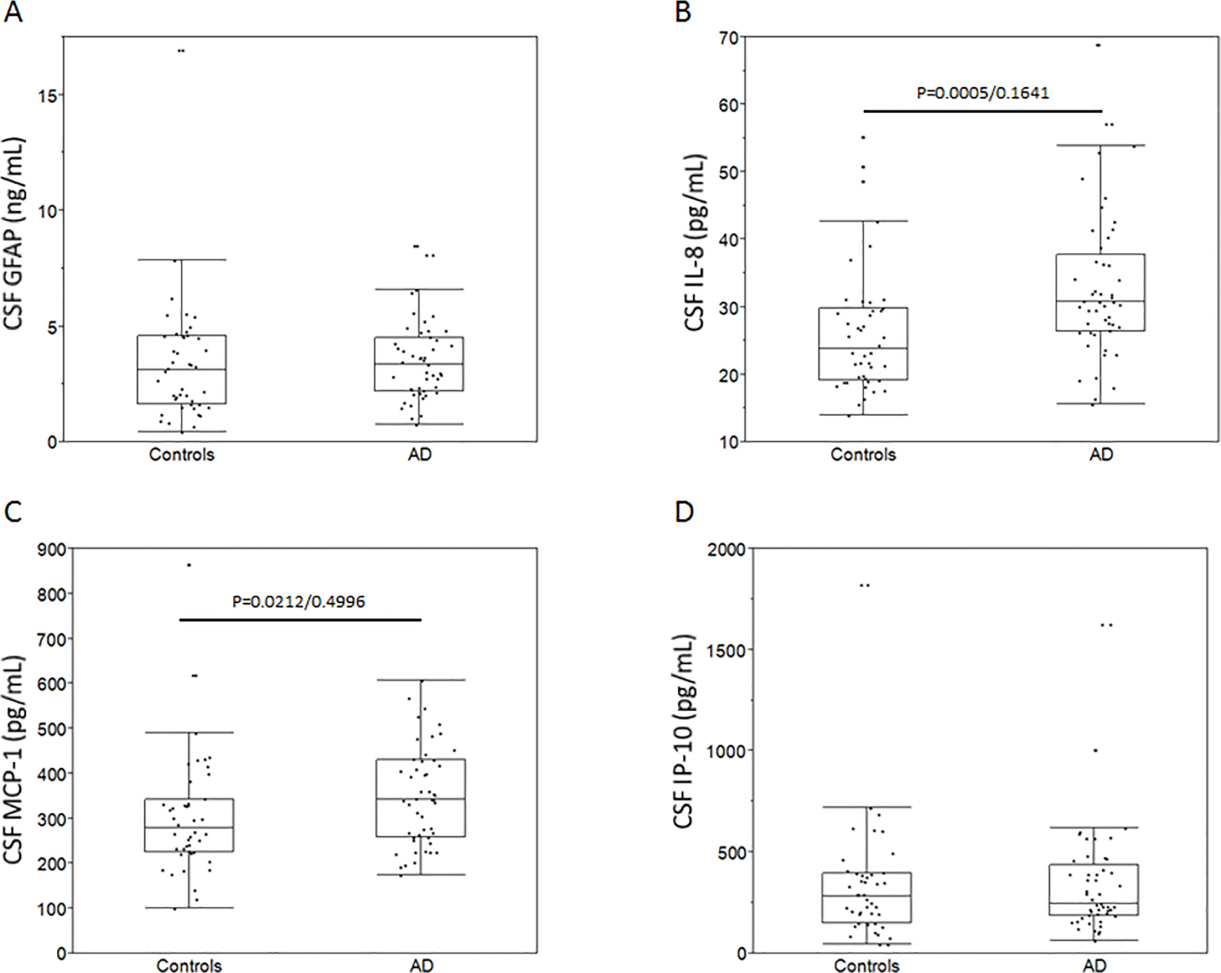
Cerebrospinal fluid levels of inflammatory markers in AD. Cerebrospinal fluid levels of the astrogliosis marker GFAP did not differ between AD patients and non-demented controls (A). Levels of both IL-8 (B) and MCP-1 (C) but not IP-10 (D) appeared increased in AD patients compared to non-demented controls. However, upon adjustment for age, significance was lost as assessed using log-transformed data and the ANCOVA test with age entered as a co-variant (displayed data are unadjusted). P-values are reported as unadjusted/adjusted.

CSF GFAP levels did not differ between AD patients and non-demented controls (Fig 3A). However, a significant difference was found between male and female control subjects where males exhibited slightly higher mean CSF GFAP levels compared to females (3.87 (1.51–16.93) pg/mL versus 2.04 (0.42–6.2) pg/mL, p = 0.0241). These differences were not present in the AD group and thus the unequal gender distribution in this particular group did not impact the outcome of the comparison between AD patients and non-demented controls. Out of the proinflammatory chemokines, levels of MCP-1 and IL-8 were elevated in the AD group versus non-demented controls (Fig 3B and 3C) however when correcting for age, these differences were not detectable anymore. We further found no differences in the CSF levels of IP-10, between non-demented controls and AD patients (ANCOVA, p = 0.0952) (Fig 3D). Levels of the inflammatory markers were not influenced by APOE genotype or gender.

### Inflammatory marker correlations to cognition and disease biomarkers

We found no association between CSF YKL-40 levels and cognition in non-demented controls, AD and DLB patients. However, a rather weak but significant negative correlation to MMSE test scores in the PD group (Spearman’s rho = -0.2787, p = 0.0296) (S2A Fig). In order to explore associations between YKL-40 levels and markers of AD pathological processes we evaluated potential links between YKL-40 and the AD biomarkers T-tau, P-Tau and Aβ1–42. Our multivariate correlation analysis showed that whereas CSF YKL-40 levels were unrelated to CSF Aβ1–42 levels irrespective of diagnostic groups, they were significantly associated with increased levels of tau in several groups. In the non-demented groups, including non-demented controls and PD patients, increased levels of T-tau were correlated to increased levels of YKL-40 (non-demented controls: Spearman’s rho = 0.5109, p = 0.0017; PD: Spearman’s rho = 0.4881, p = 0.004). Increased levels of YKL-40 were also positively linked to P-tau levels in non-demented controls (Spearman’s rho = 0.5255, p = 0.006), DLB (Spearman’s rho = 0.3665, p = 0.033) and PD (Spearman’s rho = 0.3346, p = 0.0153) but not AD patients (for distribution see S2B and S2C Fig). With decreased CSF levels of α-synuclein demonstrated as a marker of synucleinopathy [15, 20–22] we also investigated potential relationships between CSF YKL-40 and the previously reported CSF α-synuclein levels [15]. Concentrations of CSF YKL-40 were positively associated with α-synuclein in non-demented controls (Spearman’s rho = 0.4824, p = 0.0009), AD (Spearman’s rho = 0.4166, p = 0.0032), DLB (Spearman’s rho = 0.4934, p = 0.0041) and PD patients (Spearman’s rho = 0.4474, p = 0.0003) (for distribution see S2D Fig).

As YKL-40 was proposed to be produced by reactive astrocytes [7, 12, 23] we also investigated whether levels of YKL-40 and the astrocyte-specific marker GFAP, were associated. Surprisingly, we only found an association in non-demented controls (Spearman’s rho = 0.3119, p = 0.0393). No significant links were found between CSF levels of GFAP and MMSE scores, nor with AD biomarkers T-tau, P-tau and Aβ1–42 (data not shown).

We were unable to identify any associations between CSF levels of YKL-40, IL-8, IP-10 and MCP-1 in AD patients (data not shown). Additionally, neither IP-10, MCP-1 or IL-8 was associated with MMSE test scores, AD biomarkers or α-synuclein in these patients.

## Discussion

Numerous studies have evaluated various fluid biomarkers to facilitate the diagnosis of AD and to enable monitoring of disease progression in these patients [6]. There is still however a critical need for reliable clinical AD biomarkers to distinguish AD from other related disorders [24]. In the current study we set out to assess the disease-specificity of elevated CSF YKL-40 levels in AD patients, which were previously reported by various groups [7–10]. We confirm that CSF levels of YKL-40 are positively correlated to age and increased specifically in AD patients with mild to moderate dementia when compared to non-demented controls and patients with PD or DLB. Assessment of CSF YKL-40 as a clinical biomarker to enable disease discrimination by ROC curves resulted in similar AUC numbers when comparing a mild to moderate AD diagnosis to controls and a diagnosis of PD or DLB. Hence increased CSF YKL40 levels in the AD group could aid discrimination from controls and a diagnosis of PD or DLB in a similar manner. A previous study proposed decreased levels of YKL-40 in CSF from patients with PD patients [25], however we found no decrease in YKL-40 levels in the two synucleinopathy disorders included in the current study. Another small study reported unaltered levels of CSF YKL-40 levels in AD patients compared to patients with other dementias and control subjects [26]. Importantly, Mattsson and colleagues merged the controls with individuals afflicted by stable MCI for the comparison with AD patients, which complicates the interpretation of their results. Additional studies are needed to elucidate the reasons for the observed discrepancies.

Our results further showed that YKL-40 levels were not associated with Aβ42 whereas CSF tau levels were positively correlated to YKL-40 levels in all groups except for the AD group. These results suggest that YKL-40 may in fact correlate with subtle changes in tau before dementia symptoms develop and similar to the case with CSF Aβ1–42 and tau levels, reach a plateau when the disease has manifested itself. This assumption would explain the absence of increased YKL-40 levels in the DLB group which in our cohort exhibited lower Aβ1–42 levels than non-demented controls but no increase in tau levels. Further, levels of α-synuclein, which we recently reported to be increased in CSF from AD patients but decreased in patients with synucleinopathy [15], were positively associated with YKL-40 levels in all groups. As we have earlier demonstrated strong associations between CSF levels of tau and α-synuclein [15, 22], our current findings showing significant correlations between YKL-40 and α-synuclein in all investigated groups were not surprising given the previously mentioned links between tau and YKL-40.

Since reactive astrocytes have been proposed as the main source of YKL-40 in the brain we also analyzed the CSF levels of GFAP but found no differences between the investigated groups and also no significant associations with the described levels of YKL-40. Results from a study performed on AD patients that were roughly 10 years younger than the group included in the current study proposed significantly increased levels of CSF GFAP compared to controls[27]. Notably, the authors also did not report any association between age and CSF GFAP levels. We speculate that the discrepancies between the herein reported results and the ones reported by Jesse and colleagues may lie in the age difference between the investigated AD cohorts as it is not known at what age an association between age and increased inflammatory markers develops. Further, a cause for the discrepancies could also be sought in technical parameters, for instance it is not clear how GFAP levels in CSF behave upon centrifugation. Opposite to the sample handling for the current study, Jesse and coauthors did not specify any centrifugation step after CSF collection. Interestingly, a previous study looking into the tissue expression of YKL-40 and GFAP using *in situ* hybridization and immunohistochemistry demonstrated a positive association between GFAP immunostaining and YKL-40 transcription in various neurological disorders [12]. This association could not be detected in our analysis of CSF GFAP and YKL-40 concentrations in AD patients. Although GFAP is an intracellular intermediate filament protein recent studies suggest that GFAP can be released into the extracellular space for example upon trauma [28]. It is unclear however whether YKL-40 and GFAP release can be triggered by similar stimuli. We speculate that protein concentrations of GFAP and YKL-40 (a secreted glycoprotein) in the CSF do not accurately reflect their relative tissue concentrations.

To further investigate whether an increase in YKL-40 is associated with altered levels of other inflammatory markers we used a multiplexed MSD assay to quantify a panel of inflammatory markers in the AD group compared to non-demented controls. With detectable levels found only for IP-10, MCP-1 and IL-8, we found no significant differences between AD patients and non-demented controls when correcting our analysis for age. As an inherent issue with most immuno-based assays and a limitation of the current study is the inter-assay variation which for some of the analytes was rather high leading to the possibility that true biological differences may in fact be obscured. Interestingly, a previous study on age-matched control and patient groups with an average age of 62 years showed increased levels of MCP-1 and IL-8 in AD patients whereas only a subset of the studied AD patients exhibited increased levels of IP-10, compared to controls [29]. In another study by the same authors CSF levels of MCP-1, IL-8 and IP-10 were determined in patients with MCI, mild and severe AD. They found that IP-10 levels were increased in patients with MCI and mild but not severe AD. In the same study, the authors showed that MCP-1 and IL-8 were increased in both MCI and AD patients. Similar to our findings the authors reported a significant and positive correlation between age and CSF levels of MCP-1 and IL-8. However, the effect of age on these parameters was not corrected for in their statistical analyses [30]. Importantly, we previously described a correlation between higher CSF MCP-1 levels and faster cognitive decline in prodromal AD patients despite no significant differences in MCP-1 levels compared to controls at baseline[31]. Hence, the discrepancies between the results presented in the current study and the studies mentioned above could probably be explained by differences in age and disease severity of the studied subjects or the fact that the individuals included in the previous as well as the current study were clinically diagnosed which introduces a small uncertainty that may affect the outcome.

Interestingly, the herein observed increase in YKL-40 was not significantly correlated with levels of IL-8, IP-10 or MCP-1 suggesting that alternative mechanisms other than those orchestrating levels of MCP-1 and IL-8 may be controlling the levels of YKL-40 in the brain. Also, as IL-8, MCP-1 and IP-10 can be secreted by various cell types including endothelial cells[32, 33], astrocytes [34], pericytes[35, 36], microglia[37], oligodendrocytes[38, 39] and to some extent by neurons[40, 41] the overall quantified levels of those markers may as a pool from various sources not appropriately reflect the activation of specific cell populations. Of note, interleukin-1b (IL-1b) and tumor necrosis factor-α (TNF-α) were recently shown to be the main inducers of YKL-40 secretion in primary human astrocytes [23] and whilst many proinflammatory markers including MCP-1 [42] and IL-8 [43] are positively regulated by NF-κB signaling, IL-1b is decreased upon NF-κB activation [44]. Also, Rehli and colleagues previously reported an important regulatory role of transcription factor Sp-1 in the regulation of YKL-40 expression by peripheral macrophages [45]. Thus, we speculate that the observed increase in YKL-40 levels in the CSF from AD patients is an early event which may plateau or even decline once a robust NF-κB activation takes over at later stages of the disease. In support of this hypothesis, several studies have shown that the levels of YKL-40 increase at very early stages of AD [7–9]. Most recently, Antonell and colleagues reported increased CSF levels of YKL-40 in preclinical and prodromal AD proposing YKL-40 as a suitable marker for early pathophysiological changes potentially linked to neurodegenerative processes[46]. The AD patients included in our current study were suffering from mild to moderate dementia as indicated by the average MMSE total score of 20 and we acknowledge that including MCI patients and AD subjects with more severe dementia would have been beneficial to the overall conclusions of our investigation.

In summary, the herein presented results describing significantly increased levels of YKL-40 in AD patients compared to non-demented controls and patients with PD or DLB, confirm and extend previous studies suggesting CSF YKL-40 as a clinical biomarker candidate for the differential diagnosis of AD dementia. With no detectable associations with CSF levels of GFAP, MCP-1, IP-10 and IL-8 in the AD group we speculate that an induction of YKL-40 levels in AD occurs early and plateaus when dementia symptoms appear.

## Supporting Information

S1 Fig Distribution of cerebrospinal fluid AD biomarkers and α-synuclein levels. Cerebrospinal fluid levels of Aβ1–42 (A), T-tau (B), P-tau (C) and α-synuclein (D) in controls and patients with AD, DLB or PD.
(TIF)

S2 Fig Associations between cerebrospinal fluid YKL-40 levels, AD biomarkers and α-synuclein. Cerebrospinal fluid levels of YKL-40 were significantly associated with; MMSE total test scores in PD patients only (A), T-tau levels in non-demented controls and PD patients, P-tau levels in all groups except for AD patients (C) and α-synuclein levels in all diagnostic groups (D). Associations between YKL-40 levels, MMSE scores, AD biomarkers and α-synuclein levels were assessed using the non-parametric Spearman’s Rho correlation test
(TIF)
