# Supplementary figures and images for: The Inflammatory Marker YKL-40 Is Elevated in Cerebrospinal Fluid from Patients with Alzheimer’s but Not Parkinson’s Disease or Dementia with Lewy Bodies
Source: PLoS One. 2015 Aug 13;10(8):e0135458. doi: 10.1371/journal.pone.0135458 (PMC4536228; doi:10.1371/journal.pone.0135458)

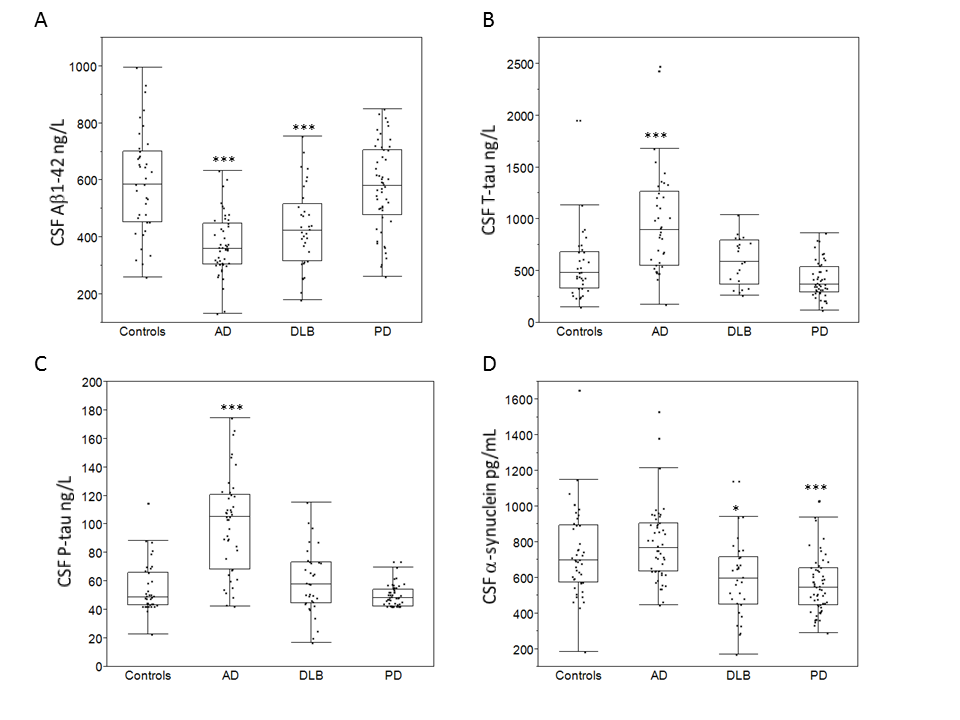

Supplement: S1 Fig — Cerebrospinal fluid levels of Aβ1–42 (A), T-tau (B), P-tau (C) and α-synuclein (D) in controls and patients with AD, DLB or PD. (TIF) [file pone.0135458.s001.tif]

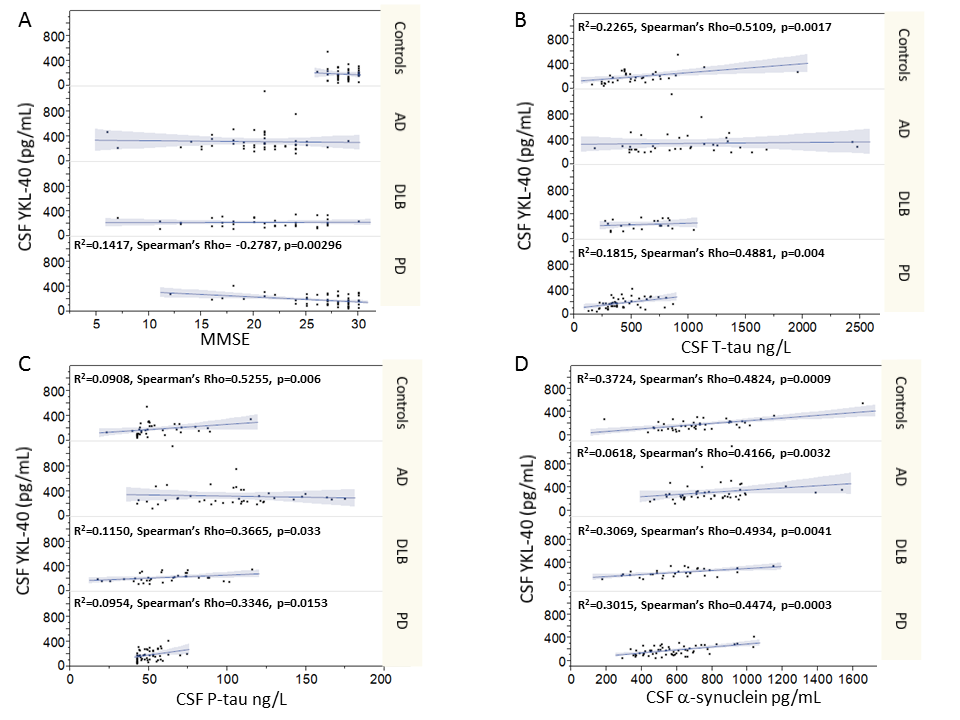

Supplement: S2 Fig — Cerebrospinal fluid levels of YKL-40 were significantly associated with; MMSE total test scores in PD patients only (A), T-tau levels in non-demented controls and PD patients, P-tau levels in all groups except for AD patients (C) and α-synuclein levels in all diagnostic groups (D). Associations between YKL-40 levels, MMSE scores, AD biomarkers and α-synuclein levels were assessed using the non-parametric Spearman’s Rho correlation test (TIF) [file pone.0135458.s002.tif]
